# Randomized control trial evidence for the benefits of massage and relaxation therapy on sleep in cancer survivors—a systematic review

**DOI:** 10.1007/s11764-020-00972-x

**Published:** 2020-12-02

**Authors:** Stephen Rajan Samuel, Rachita Gururaj, K. Vijaya Kumar, Prina Vira, P. U. Prakash Saxena, Justin William Leslie Keogh

**Affiliations:** 1 Department of Physiotherapy, Kasturba Medical College, Mangalore, Manipal Academy of Higher Education, Manipal, India; 2 Department of Radiation Oncology at Kasturba Medical College, Mangalore, Manipal Academy of Higher Education, Manipal, India; 3 Faculty of Health Sciences and Medicine, Bond University, Gold Coast, Australia; 4 Human Potential Centre, AUT University, Auckland, New Zealand; 5 Cluster for Health Improvement, Faculty of Science, Health, Education and Engineering, University of the Sunshine Coast, Sunshine Coast, Australia

**Keywords:** Massage, Relaxation therapy, Sleep wake disorders, Carcinoma

## Abstract

**Purpose:**

Cancer survivors may experience sleep disturbances during and after their cancer treatments. While pharmacological approaches are commonly used to address sleep disturbances, they may have a number of adverse effects. This review studied the effect of two non-pharmacological interventions (massage and relaxation therapy) on sleep disturbances in cancer survivors.

**Methods:**

A search for randomised controlled trials (RCTs) was conducted on PubMed, Scopus, Web of Science, PEDro, and CINAHL using relevant keywords.

**Results:**

The search yielded 371 articles, with 4 RCTs studying massage therapy and 3 RCTs studying relaxation therapy included for qualitative analysis. Massage therapy studies showed statistically significant improvement in self-reported sleep questionnaires and objectively recorded long sleep episodes, as assessed via an accelerometer. No significant improvements in sleep outcomes were observed in the relaxation therapy studies, although there were trends for improved self-reported sleep quality.

**Conclusion:**

While massage therapy provided by massage therapists may have some potential for improving sleep outcomes for cancer survivors, there is no such current evidence regarding relaxation therapy.

**Implications for Cancer Survivors:**

Cancer survivors who experience sleep disturbances may benefit from regular sessions with a massage therapist. However, future studies should examine the long-term feasibility of massage therapist–delivered services, particularly for cancer survivors with limited finances, and determine if benefits can be obtained if massage is provided by non-certified individuals. Relaxation therapy appears to be safe for cancer survivors, but future RCTs involving larger sample sizes need to be conducted to better determine its feasibility and efficacy.

## Introduction

Sleep disturbance is one of the most distressing symptoms experienced by cancer survivors not only at the time of diagnosis and treatment but also affecting 51% of cancer survivors beyond 5 years post-treatment [1, 2]. As such, the prevalence of sleep disturbances in cancer survivors is nearly twice that found in the general population [3]. Long-term sleep disturbances can lead to distress, increased morbidity, reduced productivity, and poor quality of life [1, 4], with these disturbances in cancer populations having been attributed to a variety of factors. These may include anxiety due to the cancer diagnosis, cancer-related pain, and the direct or indirect side effects of their cancer treatments (nausea, vomiting, or hot flashes) [5, 6]. Sleep disturbances in individuals with cancer may be even more exacerbated in those with cancer-related fatigue, which is another common symptom in this population [7, 8]. Similar to the general population with sleep disturbances, a pharmacological approach is typically one of the most common options for treating sleep disturbances in cancer survivors, especially in the acute stages [9, 10]. But as sleep disturbances in cancer survivors are typically a chronic problem, non-pharmacological treatments may be beneficial as they will reduce the potential adverse outcomes associated with long-term pharmacological usage.

Cognitive behavioral therapy (CBT) is currently considered the non-pharmacological treatment of choice for sleep disturbances in many populations including cancer survivors [11, 12]. Exercise is another non-pharmacological treatment with emerging systematic review–level evidence regarding its efficacy in improving sleep outcomes in cancer survivors [13, 14]. However, due to potential limitations in the accessibility and cost for supervised CBT and exercise therapy for cancer survivors living in rural/remote areas and/or with limited financial savings, there is a need to determine if therapies that have shown to be beneficial for improving sleep in other populations may also be beneficial in cancer survivors. Massage and relaxation therapy could both be examples of such non-pharmacological techniques that can improve sleep outcomes in other populations and may be more accessible to many cancer survivors than supervised CBT or exercise therapy sessions and utilized even in patients with advanced cancer.

Clinical massage refers to “the use of manual manipulation of the soft tissues to relieve specific complaints of pain and dysfunction” [15]. Swedish massage and deep tissue massage are the two most common forms of therapeutic massage, with Swedish massage more commonly used for relaxation [16] and deep tissue massage to alleviate pain [17]. Common techniques used within these massage forms may include effleurage, petrissage, percussion, myofascial release, trigger point therapy, deep transverse friction, compression massage, and cross-fiber massage. Therapeutic massage has shown to significantly improve rapid eye movement latency and stage 1 sleep and increase sleep stages 3 and 4 in postmenopausal women [18]. In patients with fibromyalgia, therapeutic massage significantly improved self-reported sleep quality [19]. Within cancer population, there is also a non-randomized controlled trial that suggests therapeutic massage is beneficial in reducing pain, boosting mood, and promoting relaxation in cancer survivors [20].

Relaxation therapy is another commonly practiced non-pharmacological treatment option that may be useful for the management of a variety of physical and psychosocial issues, including sleep disturbances. This form of therapy typically consists of breathing exercises, somatic relaxation, stretching and relaxing of the muscles in a continuous and systematic pattern to aid relaxation of the body and mind. Relaxation therapy given as a home program for patients with chronic obstructive pulmonary disease has shown to improve sleep quality [21]. Furthermore, a meta-analysis indicates that relaxation therapy has proven to be efficacious in cancer-related fatigue, especially during cancer treatment [22].

Though there are studies addressing massage and relaxation therapy for treatment of sleep disturbances in the adult and pediatric cancer population, there has been no systematic summary of the data available. Summarizing the findings of randomized control trials (RCTs) would be beneficial in developing treatment strategies for sleep disturbances in cancer survivors, during and after cancer treatment and in highlighting potential gaps in the literature that future research may look to examine. Therefore, this systematic review was performed to identify relevant RCTs to determine whether there is currently any evidence to support the use of therapeutic massage or relaxation therapy for improving sleep outcomes in cancer survivors.

## Methods

### Search strategy

A comprehensive data search was performed on PubMed, Scopus, Web of Science, PEDro, and CINAHL from inception to September 2020. The search terms used for cancer were the following: Carcinoma (MeSH), Cancer, Neoplasms (MeSH), Malignancy and Tumor. Sleep (MeSH), sleep quality, sleep disturbances, Insomnia, Sleep wake disorders (MeSH), Sleep initiation and maintenance disorders (MeSH) and Sleep disorders were the search terms related to sleep disturbance. For the interventions, Relaxation techniques, Relaxation therapy (MeSH) and Massage (MeSH) were the terms used. The search terms were combined with Boolean operator ‘AND’ or ‘OR’ wherever relevant. The reference list of the included articles was also screened for potentially relevant studies. The search was limited to RCTs involving human participants that were published in English.

### Selection of studies

After the results from each database were compiled and duplicates removed, title and abstracts were screened independently by 2 reviewers (RG and PV). Any disagreements were sorted by discussion with SRS. The screening for eligible articles was done based on the pre-set criteria. To be included in the systematic review, studies had to (1) include cancer survivors of any ages, type of cancer, or treatment status; (2) have massage or relaxation therapy as the primary intervention; (3) include comparisons to usual care or active/sham treatments; (4) report self-reported or objectively measured sleep outcomes; and (5) involve RCT designs, with the results reported in peer-reviewed journals. Cancer survivors were defined according to the National Cancer Institute definition that states “a person is considered to be a survivor in cancer from the time of diagnosis until the end of life” [23]. Studies reporting any form of massage such as deep friction massage, Swedish massage, and its components used for therapeutic benefit were included in the review. Studies evaluating relaxation therapy techniques used independently, consisting of breathing exercises, somatic relaxation, and progressive relaxation techniques were included in the review. However, other forms of complimentary treatments that included relaxation therapy as a part of the treatment were excluded.

### Data extraction and management

All the eligible studies were screened further based on full text independently by RG and PV. Any disagreements were sorted after discussion with SRS, VK, and JK. A data extraction template was developed for the eligible studies which included information on the demographic characteristics of the participants, description of the intervention, comparator, treatment status, and key findings. Data extraction was performed by RG and PV and disagreements were sorted by discussions with SRS, VK, and JK.

### Assessment of risk of bias

The assessment of risk of bias for the included studies was performed independently by RG and PV using the Cochrane risk of bias tool [24]. The studies were scored under the domains of random sequence generation, allocation concealment, blinding of participants and personnel, blinding of outcome assessment, incomplete outcome data, selective reporting, and other bias. The judgment of yes was marked as low risk and no as high risk. Unclear was marked if the details were missing or unknown bias. Any disagreements in the markings between the 2 reviewers were sorted by discussions with SRS.

## Results

### Characteristics of studies

**Fig. 1:**
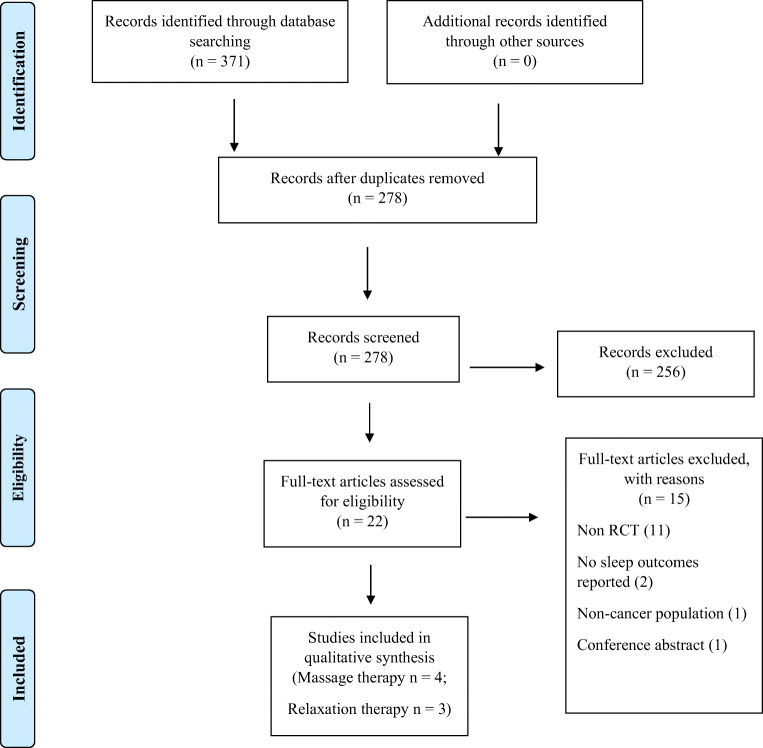
PRISMA flowchart describing the study selection process

As seen in the PRISMA flowchart (Fig. 1), a total of 371 articles were retrieved from the data search (PubMed-77, Scopus-159, Web of Science-78, CINAHL-2, and PEDro-55). A total of 278 articles were screened based on title and abstract after removing the duplicates. Of the 22 articles that were found to be potentially eligible and underwent full-text screening, 7 articles which met the inclusion criteria were included in the review. The most common reasons for exclusion of the articles included non-randomized controlled trial methodologies and a lack of reported sleep outcomes.

### Risk of bias

**Fig. 2:**
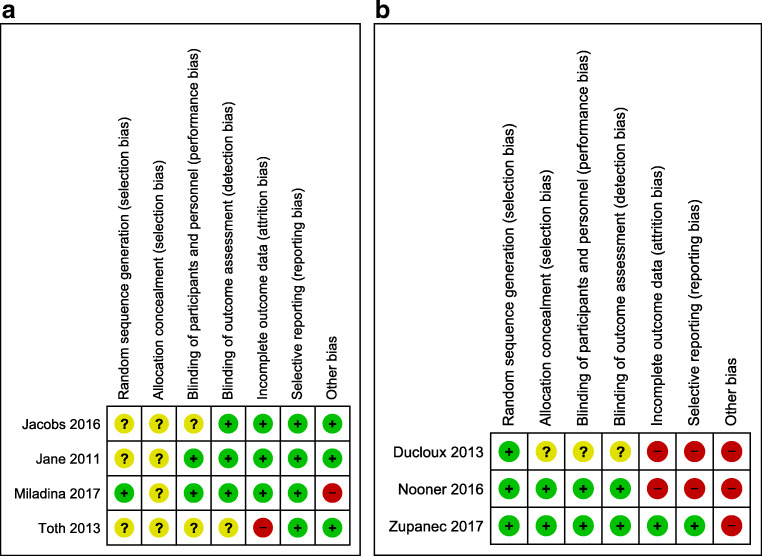
**a** Risk of bias of massage therapy studies. Green - low risk, yellow - unclear risk, and red - high risk of bias. **b** Risk of bias of relaxation therapy studies

The summary of the risk of bias assessments of the included studies has been represented in Fig. 2a and b. Of the 4 massage therapy studies, a high risk of bias was reported for Incomplete Outcome Data [25] and Other Biases [26]; with unclear risk of bias reported for Random Sequence Generation [25, 27, 28], Allocation Concealment [23, 26–28], Blinding of Participants [25, 27], and Blinding of Outcome Assessment [25]. Of the 3 relaxation therapy studies, a high risk of bias was reported for Incomplete Outcome Data, Selective Reporting [29, 30], and Other Biases [29–31]; with unclear risk of bias reported for Allocation Concealment [29], Blinding of Participants [29], and Blinding of Outcome Assessment [29].

### Population of included studies

Among the 7 included studies, 6 studies involved adults from 18 to 78 years [25, 27–30] and 1 involved pediatric population with an age range of 4–8 years [31]. Four studies included participants with mixed type of cancers [25, 27, 29, 30], 3 studies included participants with metastatic cancer [25, 28, 29], and 2 studies included participants with leukemia [26, 31].

### Intervention of included studies

Four out of 7 RCTs studied the effect of massage therapy [25–28], with the other 3 examining relaxation therapy [29–31] as their intervention. The total number of participants recruited for the 4 RCTs studying the effect of massage therapy was 205, out of which 187 completed the entire study. There were 50 participants recruited across the 3 RCTs studying the effect of relaxation therapy, with 33 participants completing the study.

### Massage therapy

The 4 included RCTs that studied the effect of massage therapy, delivered either by massage therapists or nurses trained in massage, are summarized in Table 1. The duration of the interventions ranged from 2 days to 4 weeks, involving 2 to 3 massage sessions per week, with each massage therapy session ranging from 10 to 45 min in duration. Two studies compared the effect of massage with usual care [26, 27], 1 study compared the intervention to a social attention control [28], and the remaining study compared massage with usual care and no-touch control interventions [25]. One study used a MiniMotionlogger actigraph with sleep diary to objectively measure sleep [27], whereas the remaining 3 studies used self-reported questionnaires including the Richards-Campbell Sleep Questionnaire (RCSQ) and Pittsburg Sleep Quality Index (PSQI) to assess sleep quality [25, 26, 28].

**Table 1:** Summary of massage therapy studies

Reference	Sample			Intervention details		Results	
	Cancer type and stage	Age, sex, and sample size	Cancer treatment and sleep disorder status	Description	Duration and frequency	Pre	Post
Jane et al. 2011 [28], Taoyuan, Taiwan	Mixed cancer type, stage 4 patients with metastatic bone cancer	50.1 ± 11.5 yr (15 M, 21 F)	Cancer treatment: completed primary treatment, with 56% under palliative care; sleep disorder: not stated	Effleurage, light petrissage, compression, and nerve stroke to total body, provided by cancer nurses with 3 months of massage training.	45 min for 3 days	RCSQ (T1): 22.1 ± 15.1	RCSQ (T3): 32.8 ± 11.4^#^
		49.7 ± 9.7 yr (15 M, 21 F)		Social attention encouraging participants to express feelings or concerns about admission involving the presence of a caring therapist		RCSQ (T1): 25.1 ± 13.6	RCSQ (T3): 30.2 ± 14.4
Toth et al. 2013 [25], Boston, USA	Mixed (stage 4) patients with metastatic cancer	54.9 ± 12 yr (3 M, 17 F)	Cancer treatment: not stated; Sleep disorder: not stated.	Swedish and non-Swedish massage techniques including gliding/effleurage, light petrissage, compression, and nerve stroke to non-metastatic areas provided by professional massage therapists	15–45 min/day, 3 daysa week for 1 week		RCSQ CS (1 week): − 3.5 (− 9, 0) RCSQ CS (1 month): − 4 (− 12, 0)
		54.9 ± 10 yr (1 M, 9 F)		No-touch control provided by professional massage therapists with hands 12 in. (30 cm) above participants but with no healing intention			RCSQ CS (1 week): 0 (− 7, 9) RCSQ CS (1 month): 0 (− 12, 10)
		55.6 ± 9 yr (3 M, 6 F)		Usual care participants received no visits from massage therapists			RCSQ CS (1 week): − 0.5 (− 4, 1) RCSQ CS (1 month): 0 (− 15, 12)
Jacobs et al. 2016 [27], Washington, USA	Mixed type and stage with at least 4 days of hospitalization	15.5 ± 2.6 yr (12 M, 4 F)	Cancer treatment: 70% receiving chemotherapy. Sleep disorder: not stated.	Swedish massage of the total body by massage therapists	20–30 min/day for 2–3 days	Pre- (outcome mean) SMN: 366 SEN: 59 WM: 259 WE :18 LSE: 11 24SM: 391 24SE: 13	Post- (outcome mean) SMN: 419 SEN: 64 WM: 258 WE: 19 LSE: 12* 24SM: 444 24SE: 17
		16 ± 2.5 yr (12 M, 6 F)		Usual care received no special treatment other than the primary chemotherapy treatment.		SMN: 379 SEN: 63 WM: 247 WE: 20 LSE: 13 24SM: 431 24SE: 18	SMN: 365 SEN: 67 WM: 194 WE: 16 LSE :11 24SM: 401 24SE: 16
Miladina et al. 2017 [26], Alwaz, Iran	Acute leukemia undergoing chemotherapy	33.9 ± 9.6 yr (16 M, 14 F)	Cancer treatment: chemotherapy; Sleep disorder: ≥ 3 on Numerical Rating Scale and > 5 on PSQI	Slow-stroke back massage involving circular, sweeping hand strokes extending from the skull to sacrum by oncology nurses with 4 months of massage training.	10 min/day, 3 days a week for 4 weeks	PSQI Pre: 12.23 ± 3.75	PSQI Post: 9.70 ± 3.27*
		35.1 ± 9.6 yr (15 M, 15 F)		Usual care routine nursing and medical care		12.10 ± 3.45	12.37 ± 3.43

*CS* change score, *F* females, *LSE* long sleep episodes at nighttime, *M* males, *PSQI* Pittsburgh Sleep Quality Index, *RCSQ* Richards-Campbell Sleep Questionnaire, *SEN* sleep efficiency at nighttime, *SMN* sleeping minutes at nighttime, *t1* first session, *t3* third session, *WE* wake episodes during sleep at nighttime, *WM* wake min after sleep onset at nighttime, *yr* years, *24SE* long sleep episodes through whole day, *24SM* sleeping minutes through the whole day

*Statistically significant (*p* < 0.05) between-group improvement

^#^Statistically significant (*p* < 0.05) within-group improvement

Significant improvements in self-reported sleep quality were reported in 2 studies, with this including a significant between-group improvement in PSQI [26] and a trend for a significant between-group improvement in RCSQ (*p* = 0.07) after the first day of the massage intervention [28]. The only significant benefit observed for any objective sleep outcomes was reported in a study utilizing the Motionlogger device, whereby Jacobs et al. [27] observed a significant between-group increase in the number of nighttime long sleep episodes for the massage compared to the control group.

Feasibility of the massage interventions were reported in the studies conducted by Toth et al. and Jacobs et al. [25, 27]. Toth et al. [25] reported that it was feasible to provide in-home massage therapy to the participants by professional massage therapists, with each participant averaging 2.8 sessions of the required 3 sessions during the 1-week trial. In the study of Jacobs et al. [27], the goal was for massage therapists to provide 2–3 massages to adolescent patients with cancers who were staying in the hospital for a minimum of 4 days. Results indicated reasonable feasibility, in that 94% of the participants received 1 massage and 69% received 2 massages during their hospital stay. While feasibility outcomes were not directly assessed in the other studies, some indication of the feasibility for the other 2 studies may be provided by comparing the number of participants initially recruited with the number of participants who completed the study. Of the 72 participants recruited in the study by Jane et al., 2 and 3 dropped out by the second session (T2) in the massage and social attention group, respectively [28]. In the study conducted by Miladinia et al., out of the 64 recruited participants, only 3 participants in the massage therapy group and 1 in the usual care group were lost to follow-up [26].

The safety of these massage interventions was examined by monitoring the number of adverse events in 2 of the 4 studies. No adverse events related to the massage therapy were reported in either of these studies [25, 28].

### Relaxation therapy

As summarized in Table 2, there were 3 RCTs that studied the effect of relaxation therapy on sleep disturbances in cancer populations [29–31]. All 3 RCTs were pilot studies with the duration of intervention lasting from 3 to 60 days that examined if a variety of sleep outcomes could be improved compared to usual care. Two out of the 3 studies provided some degree of relaxation guidance by the therapist, with 1 study appearing to only provide relaxation resources and written instructions [30]. The outcome tools used 3 self-reported questionnaires including the Numerical Rating Scale of Satisfaction of Sleep (NRSSS), Patient-Reported Outcomes Measurement Information System (PROMIS), and the Children Sleep Habits Questionnaire (CSHQ) as well as objective sleep data from the accelerometers including the Minimotionlogger actigraph.

**Table 2:** Summary of relaxation therapy studies

Reference	Sample			Intervention details		Results		
	Cancer type and stage	Age, sex and sample size	Cancer treatment and sleep disorder status	Description	Duration and frequency	Pre	Mid	Post
Ducloux et al. 2012[29], Geneva, Switzerland	Mixed (stage 4) with metastatic cancer and estimated prognosis < 6 months	61 ± 15 yr (3 M, 6 F)	Cancer treatment: not stated; Sleep disorder: diagnosed using ICSD criteria.	Immediate intervention: Deep breathing exercises, somatic tension release was taught by a specialized nurse with certification in relaxation. An audio recording of the program on CD was provided to the participants to use prior to nighttime sleep.	1 h/day for 3 days	NRSSS day 1: 6.1 + 2.4	NRSSS day 2: 3.6 ± 2.3	NRSSS day 5: 4.0 ± 2.3
		66 ± 12 yr (3 M, 6 F)		Delayed intervention: as above, but started 3 days after the immediate intervention group.		6.5 + 2.2	4.0 ± 2.1	3.8 ± 2.3
Nooner et al. 2016 [30], OK, USA	Hematologic malignancies or solid tumors, undergoing chemotherapy or prior to HSCT	45 ± 18 yr (6 M, 5 F)	Cancer treatment: chemotherapy and pre-HSCT therapy; Sleep disorder: not stated.	Guided imagery: 19-min audio program-morning exercise, which guides the listener through waking in the morning.	19 to 39 min/day for 60 days	PROMIS sleep baseline: Pt.5: 23/40 Pt.7: 19/40 Pt.9: 25/40	PROMIS sleep day 30: 21/40 17/40 29/40	PROMIS sleep day 60: - 20/40 -
				Relaxation: 39-min audio program guide to serenity which guided the listener through a 10-point system of progressive relaxation.		Pt.2: 25/40 Pt.3: 35/40 Pt.10: 27/40	20/40 - -	26/40 - -
				Relaxation + guided imagery - received both.		Pt.1: 28/40 Pt.8: 25/40 Pt.12: 17/40	- 22/40 21/40	- 20/40 19/40
				Usual care: received usual medical care but were given relaxation video resources used in the intervention after study was completed.		Pt.4: 22/40 Pt.6: - Pt.11: 20/40	21/40 - -	- - -
Zupanec et al. 2017 [31], Toronto, Canada	Children with ALL undergoing maintenance chemotherapy	6.3 ± 1.8 yr (10 M, 1 F)	Cancer treatment: Maintenance- chemotherapy; Sleep disorder: excluded from study if they had a physician- diagnosed sleep disorder such as insomnia or restless leg syndrome.	Education and children’s books to promote relaxation using deep breathing and progressive muscle relaxation; facilitated by 2 registered nurses who had completed an 8-h training course on sleep hygiene and relaxation techniques	4 weeks	NS: 456 ± 105 LSNS: 120 ± 32 DS: 42 ± 27 LSDS: 28 ± 19 WTASO: 117 ± 44 NNA: 16 ± 4 CSHQ: 50 ± 8	-	NS: 498 ± 65 LSNS: 129 ± 44 DS: 32 ± 51 LSDS: 17 ± 28 WTASO: should 99 ± 47 NNA: 17 ± 5 CSHQ: 48 ± 8
		6.2 ± 2.0 yr (8 M, 1 F)		Usual care - advised to continue usual clinical activities.		NS: 488 ± 36 LSNS: 135 ± 28 DS: 21 ± 17 LSDS: 16 ± 12 WTASO:75 ± 39 NNA: 16 ± 3 CSHQ: 49 ± 6	-	NS: 495 ± 59 LSNS: 134 ± 89 DS: 11 ± 14 LSDS: 5 ± 7 WTASO:106 ± 43 NNA: 18 ± 6 CSHQ: 48 ± 6

*ALL* acute lymphoblastic leukemia, *CSHQ* Children sleep habits questionnaire, *DS* Daytime sleep, *F* females, *ICSD* International Classification of Sleep Disorders, *LSDS* longest stretch of daytime sleep, *LSNS* longest stretch of nighttime sleep, *M* males, *NNA* number of nighttime awakenings, *NRSS*S Numerical Rating Scale of Satisfaction of Sleep, *NS* Nighttime sleep, *PROMIS* Patient-Reported Outcomes Measurement Information System, *Pt* participant, *WTASO* wake time after sleep onset, *yr* years

□No face-to-face therapist guidance appeared to be provided to the intervention participants

No statistically significant improvements were observed in any sleep outcomes as a result of relaxation therapy, although there were some potential trends observed. Specifically, a trend for significant improvements in self-reported sleep quality was observed after the second day of relaxation training as assessed by the NRSSS [29], although no exact *p* value was provided. Sleep disturbances, as assessed by the PROMIS, were also concluded to have been improved by the authors in 2 of the 3 participants in the relaxation group and in 1 of the 3 participants in the relaxation plus guided imagery groups in the case series by Nooner et al. [30]. The authors also stated that all but 1 of these participants reported more refreshing sleep and fewer sleep symptoms after 30 days of relaxation therapy [30]. However, it must be acknowledged that none of these improvements in the Nooner et al. [30] study appears to have undergone statistical comparisons. Of the 7 objective sleep outcomes assessed using an actigraph by Zupanec et al. [31], there was a trend for an improvement in the wake time after sleep onset (*p* = 0.08) for the relaxation compared to the usual care group.

Feasibility data for relaxation therapy was provided by Zupanec et al. [32]. The 8 participant families (childhood cancer survivor and their parents) who completed the study rated the education session to be somewhat or very useful, with the sleep tips as well as the relaxation books and associated techniques reported to be the major resources used [31]. The major barriers faced related to the children’s (1) fatigue levels being so high that daytime naps were still required, with such naps potentially negatively affecting the time required for implementing the relaxation intervention; late-night medication interfering with early bedtimes; and (3) resistance to reading the same relaxation book every day [31]. Although feasibility was not directly assessed in the remaining studies, some indication of the feasibility of these interventions may also be provided by examining the number of participants who started and completed each of the relaxation therapy programs. For example, Ducloux et al. [29] recruited 9 participants into both their immediate and delayed intervention groups, with 7 participants completing the immediate intervention but only 4 completing the delayed intervention. Twenty participants were randomized to the Nooner et al. study. Nine of the 11 relaxation therapy participants and all 9 usual care participants completed the study [30]. In contrast, of the 3 participants allocated to the each of the 4 groups (relaxation, guided imagery, relaxation and guided imagery, and usual care), the relaxation group maintained only 2 participants at 30 days and 1 participant at 60 days, compared to the combined relaxation and guided imagery group who maintained 2 participants at 30 and 60 days. The dropouts in the study of Nooner et al. [30] were attributed to death, loss to follow-up due to complications of treatment, and patient request. The reasons that contributed to some participants discontinuing the Nooner et al. [30] study were described in a participant evaluation of the study. Specifically, the negative perceptions of the intervention that may have increased dropout rates were that the relaxation program may have been “too much to keep up” during complicated periods of cancer treatment and that completing the symptom log was too much work.

There appeared to be no monitoring of adverse events in any of the 3 relaxation therapy studies [29–31].

## Discussion

Cancer survivors are at a higher risk of suffering from cancer-related fatigue and sleeping disorders than members of the general population [3]. Currently, cancer survivors experiencing sleep disturbances generally undergo pharmacological treatment, with such an approach having other potential side effects [33]. The side effects of pharmacotherapy in management of sleep include drowsiness, grogginess, inability to concentrate, and difficulty in managing work and social relationships [34]. While systematic review–level evidence indicates that CBT [11] and exercise [13] are non-pharmacological treatments that can improve sleep outcomes in cancer survivors, not all cancer survivors may be interested or able to access supervised CBT or exercise therapy sessions to improve their sleep. Therefore, cancer survivors and clinicians could benefit from more evidence regarding the potential use of other non-pharmacological techniques for the management of sleep disorders.

Within the current review, 2 of the 4 studies involving massage therapy, as provided by certified therapists, observed statistically significant self-reported sleep quality (PSQI) or objective improvements in sleep outcomes (number of long sleep episodes) in cancer survivors, consistent with the findings reported for postmenopausal women and patients with fibromyalgia [18, 19]. Such improvements in sleep outcomes for cancer survivors might be of major clinical benefit, as the duration of massage therapy provided was comparatively shorter when compared to the other non-pharmacological interventions like exercise and CBT [13, 35]. Within the 4 massage therapy studies included in this review, there was relative heterogeneity in the cancer survivors as well as the type, frequency, and duration of massage therapies provided. While such variation in the massage therapy might be required to avoid massaging the tumor or metastasis sites, the heterogeneity in interventions provided make it difficult to provide any clear clinical guidelines on the optimal type, frequency, and duration of massage therapy required to improve sleep outcomes in cancer survivors. Nevertheless, the findings of this review suggest that a minimal dose of 90–120 min (be it spread out across 3 days or several weeks) of massage therapy provided by massage therapists can produce some significant benefits in sleep quality as reported by PSQI and sleep episodes as measured by accelerometers in cancer survivors. However, due to the short study duration and lack of follow-up data, the long-term effects of massage therapy in improving sleep outcomes in cancer survivors are virtually unknown.

Massage therapy, be it delivered at the cancer survivor’s home by a travelling massage therapist or within the hospital setting, appears to be quite feasible within the research studies reviewed, with most of the planned massage therapy sessions being completed and with relatively small rates of participant dropout [25–28]. However, it is unclear what proportion of cancer survivors would be able to pay for ongoing massage therapy appointments or whether the cancer survivors’ family, friends, or caregivers may be sufficiently trained to provide free and effective massage therapy to these individuals.

Among the 3 studies which studied relaxation therapy, there were no statistically significant improvements in any self-reported or objective sleep outcomes for the relaxation compared to control groups. However, there were trends for significant improvements in self-reported sleep quality, as assessed by the NRSSS [29] and for Actigraph recorded wake time after sleep onset [31]. Nooner et al. [30] also stated that all but 1 of the participants receiving relaxation training reported more refreshing sleep and fewer sleep symptoms, although no statistical data was actually provided to support these comments. The lack of significant improvements in sleep outcomes within the 3 relaxation therapy studies may have reflected a variety of between-study differences in the interventions or baseline characteristics of the participants. The relative lack of significant benefit of the relaxation therapy studies may have also been a reflection of the relatively high dropout rates in the studies. For example, while Zupanec et al. [32] described how the childhood cancer survivors and their parents thought that a number of the relaxation therapy resources and sleep tips were beneficial, they also reported many barriers such as the child’s nighttime medications and reluctance to read the same book every day to the ongoing implementation of this intervention with their children. The lack of ongoing face-to-face or telephone support of these programs by psychologists or psychiatrists and the resultant inability to tailor the relaxation therapy techniques to the individual participants’ requirements and preferences may have been some of the reasons for the relatively poor adherence and lack of significant improvements in sleep outcomes. Such views appear consistent with the wider literature on the barriers to the successful and effective utilization of relaxation therapy in clinical populations [36, 37]. Therefore, among the 7 RCTs examined, there appeared to be more support for massage therapy than relaxation therapy for improving sleep outcomes in cancer survivors. However, these preliminary conclusions require more published trials before any definitive conclusions can be drawn.

### Strengths and limitations

To the best of our knowledge, this is the first systematic review summarizing the effect of massage or relaxation therapy for sleep disturbances in cancer survivors. We used a list of comprehensive search terms developed by in-depth search and discussions among the review team, and the search was run on a wide range of databases including PEDro which is specifically for physiotherapy interventions. The observation that some of the reviewed studies consisting of participants with metastatic cancer obtained some significant benefits in sleep outcomes was of interest, as there appears to be no current RCT-level evidence regarding the effectiveness of other non-pharmacological interventions for improving sleep outcomes in cancer survivors with metastatic cancer. Furthermore, the inclusion of self-reported sleep outcomes that provided insight into the patients’ perspective was also considered a strength of the studies included in this review.

The limitations of this review process include restriction of articles to English and also the exclusion of gray literature. Unfortunately, there are also many limitations of the literature that were included in the review. Several of the included studies, especially those examining relaxation therapy, were unable to be described as low risk of bias due to the missing information reported regarding selection, performance, and detection bias. Many of the studies also did not provide detailed information regarding the actual massage or relaxation therapy intervention, which then may make replication of the intervention by practitioners or other researchers difficult. We also acknowledge that the included studies typically involved a small number of participants, cancer types, stages of cancer, and current treatments, with only one of the studies having inclusion criteria specifying that all participants needed to have been diagnosed with a sleep disorder by the International Classification of Sleep Disorders definition. With respect to the sleep outcomes, while there were some objective outcomes provided via accelerometry-derived data, such measures are indirect and not an example of outcomes recommended to be used in insomnia research and clinical practice [38]. Some relaxation therapy studies had a high dropout rate, which suggests there may be some issues in the widespread uptake of this therapy in cancer survivors. Furthermore, as many studies utilized a relatively large number of sleep outcomes but at best only found significant improvements in a small subset of these outcomes, there exists the potential for type I errors to have occurred. Finally, none of these studies utilized a follow-up period to assess the length of time the treatment effects may last post-therapy.

## Conclusion

The results of this systematic review provide some preliminary peer-reviewed support for the potential effectiveness and feasibility of massage therapy in both adult and pediatric cancer survivor populations. In contrast, the lack of statistically significant relaxation therapy-related improvements in cancer survivors’ sleep outcomes and the relatively high dropout rates in the studies included are unable to provide any evidence-based support for this therapy for cancer survivors. The results of this systematic review when viewed with respect to the limitations of the included studies would suggest that CBT and perhaps exercise should remain as the 2 primary non-pharmacological treatments for improving sleep outcomes in cancer survivors. Therefore, future massage and relaxation therapy research should look to minimize the potential risk of biases of the study designs; involve larger sample sizes of a variety of demographic groups (e.g., age, sex, countries, or cultural groups), cancer types, and treatment options; utilize more direct sleep outcomes as advocated by the Australian Sleep Association [38]; and compare the potential benefits of these therapies to more established treatments such as CBT. As relaxation therapy may also be a component of CBT, it may also be useful to design studies that are able to quantify the unique contribution of relaxation within CBT and other mind-body interventions for improving sleep outcomes in cancer survivors.

## Abbreviations

CBT: Cognitive behavioral therapy
CSHQ: Children sleep habits questionnaire
NRSSS: Numerical Rating Scale of Satisfaction of Sleep
PROMIS: Patient-Reported Outcomes Measurement Information System for Sleep - Short Form
PSQI: Pittsburgh Sleep Quality Index
RCSQ: Richards-Campbell Sleep Questionnaire
RCT: Randomized controlled trial
